# HER2-Altered Non-Small Cell Lung Cancer: Biology, Clinicopathologic Features, and Emerging Therapies

**DOI:** 10.3389/fonc.2022.860313

**Published:** 2022-03-29

**Authors:** Xin Yu, Xianxiu Ji, Chunxia Su

**Affiliations:** Department of Oncology, Shanghai Pulmonary Hospital & Thoracic Cancer Institute, Tongji University School of Medicine, Shanghai, China

**Keywords:** HER2, ERBB2, non-Small Cell Lung Cancer (NSCLC), targeted therapy, immunotherapy

## Abstract

Multiple oncogenic molecular alterations have been discovered that serve as potential drug targets in non-small cell lung cancer (NSCLC). While the pathogenic and pharmacological features of common targets in NSCLC have been widely investigated, those of uncommon targets are still needed to be clarified. Human epidermal growth factor receptor 2 (HER2, ERBB2)-altered tumors represent a highly heterogeneous group of diseases, which consists of three distinct situations including mutation, amplification and overexpression. Compared with breast and gastric cancer, previous studies have shown modest and variable results of anti-HER2 treatments in lung cancers with HER2 aberrations, thus effective therapies in these patients represent an unmet medical need. By far, encouraging efforts towards novel treatment strategies have been made to improve the clinical outcomes of these patients. In this review, we describe the biological and clinicopathological characteristics of HER2 alterations and systematically sum up recent studies on emerging therapies for this subset of patients.

## Introduction: The History of HER2 at a Glance

In the 1980s, researchers in Robert Weinberg’s laboratory isolated a cDNA clone (which was termed as Neu) from carcinogen-induced tumors and found it displayed a protein structure that highly resembled epidermal growth factor receptor (EGFR) as earlier identified. From that point, successful efforts by three independent laboratories to identify EGFR-related cDNA sequences yielded the human orthologue of Neu (now called HER2 or ERBB2) (1). However, unlike the mutation in the sequence of rodent Neu reported by Weinberg, human HER2 is usually amplified in tumors. Based on preclinical studies of breast cancer cell lines, HER2 amplification was found in a subset of patients with breast cancers and emerged as an important predictor of resistance to hormonal and chemotherapy regimens, time to relapse and overall survival (OS) (2). This significant finding was continuously confirmed by later studies and extended to gastric cancer (3).

HER2 amplification and protein overexpression lead to the dimerization of the receptor and activation of several signaling pathways that drive tumorigenesis. Consequently, targeting HER2 has been investigated as a promising therapeutic strategy. In 1998, trastuzumab, a monoclonal antibody (mAb) against HER2, was the first HER2-targeted agent approved by the FDA for treating metastatic breast cancer, which represented the beginning of a turnaround for the poor clinical outcomes of HER2-positive disease. Later on, considerable progress has been made as several categories of HER2-targeting agents, including additional mAbs, signal transduction inhibitors, novel tyrosine kinase inhibitors (TKIs) which showed excellent efficacy in both early-stage and metastatic HER2-positive breast cancer. The second malignancy suitable for trastuzumab-based therapy as a standard of care was gastric cancer. Additionally, HER2 amplification/overexpression has also been observed in other solid tumors including the biliary tract, ovarian, endometrial, bladder, colon and NSCLC (4). Disappointingly, targeting HER2 with traditional anti-HER2 agents which were proved to be effective in breast and gastric cancer has failed in other tumor types, indicating the histological and biological diversity of HER2 alternations in distinct malignancies (5).

In the subsequent sections, we will review available data and describe the biology of the HER2 pathway in normal and tumorigenesis processes, trying to provide a comprehensive overview of its dysregulation, clinical implications, as well as recent studies of emerging therapies for NSCLC patients with HER2 alterations.

## The Biology of HER2 and its Dysregulation in NSCLC

The ERBB family is comprised of four members that belong to the transmembrane tyrosine kinase receptors (TKR), including EGFR (also known as HER1), HER2, HER3 and HER4. HER2 encodes a transmembrane TKR which consists of three domains: an extracellular domain (ECD), a transmembrane domain (TMD) and an intracellular tyrosine kinase domain (TKD). Ligand binding results in heterodimerization or homodimerization between the ERBB receptors, and it sequentially stimulates the transactivation of the intracellular tyrosine kinase domain and activates downstream signaling pathways concerning cellular proliferation, differentiation, migration and apoptosis. However, HER2 lacks specific endogenous ligands and retains in the active conformation, making it continuously available for dimerization and to be the preferred heterodimerization partner. In contrast, HER3 has several ligands but lacks intrinsic tyrosine kinase activity. Interestingly, HER2-HER3 pairing displays the highest potency regarding the interaction strength and downstream signaling cascade, suggesting a complementary action between them (6–8).

HER2 is a common oncogene identified in various cancer types and dysregulation of HER2 signaling can be caused by mutation, amplification and overexpression. All three types of HER2 dysregulation could appear in NSCLC with almost no overlap between mutation and amplification (9). Thus, associations of the three types of HER2 alterations are much more complex. This phenomenon partially explains the observed poor outcomes of classic HER2-targeted therapies in the setting of NSCLC than in breast cancer, where its oncogenesis predominantly relies on HER2 overexpression attributed to gene amplification.

HER2 mutation is identified in 2%-4% of NSCLC and encompasses heterogeneous alterations distributed in the ECD, TKD and TMD. Exon 20 insertions that occurred within the kinase domain are the dominant forms of all the HER2 mutations and these insertions might account for about 1.5% of NSCLC. The most common variant is a 12-base-pair (encoding YVMA) in-frame duplicated insertion at codon 775 of exon 20, which affects the αC-β4 loop of the kinase domain and is identified as an early event in lung adenocarcinoma (LUAD) tumorigenesis (10). The HER2^YVMA^ subtype accounts for 34%-83% of HER2-mutated NSCLC, followed by G778_P780dup and G776delinsVC (11–13). In addition, there are more types of point mutations affecting the TKD but with a lower prevalence. Mutations in the TKD lead to conformational changes of ATP-binding pocket, which enhances kinase activity and downstream signaling. Other rarer mutations could also affect the ECD (mostly S310 in exon 8) and TMD (mostly V659 and G660 in exon 17) (14, 15). Generally, different mutation variants have heterogeneous behaviors, which could affect inhibitor binding affinity and sensitivity (13). In rare cases, HER2 mutations could be found in germline and cause hereditary and sporadic LUADs (16). Concomitant EGFR somatic mutations are often detected in EGFR/ERBB2 germline mutations, suggesting that patients carrying EGFR/ERBB2 germline mutations could also acquire somatic mutations in EGFR that eventually drive tumorigenesis (17).

HER2 amplification accounts for 2%-4% in NSCLC, which is far less common compared with breast cancer (18). Studies have shown that HER2 amplification and HER2 mutations were distinct molecular targets that may have different therapeutic and prognostic values. But they could co-exist in very few cases (9, 19). Though an official consensus is not available, generally HER2 amplification is defined as HER2/CEP17 ≥ 2.0 by fluorescent *in situ* hybridization (FISH) testing. Notably, it should be distinguished from HER2 copy number gain (CNG), which happens even more frequently as a consequence of chromosome 17 polysomy and is not supposed to drive tumorigenesis (9, 20, 21). Chromosome 17 polysomy leads to the increased copy number of HER-2 per cell, so as to the activity of upstream promoter, resulting in the larger expression of HER2 gene. HER2 CNG is usually defined by HER2/CEP17 < 2.0 and HER2 gene copy number ≥ 6 per cell. By far, the predictive or prognostic role of HER2 CNG in NSCLC remains unclear. Apart from *de novo* tumorigenesis, HER2 amplification is one of the most frequent acquired resistance mechanisms following the EGFR T790M mutation in EGFR-mutant NSCLC treated with first- or second-generation EGFR-TKIs, which accounts for about 10% of cases (22). It can also confer resistance to osimertinib therapy, with a lower incidence rate of 2%-5% (23). A combination of osimertinib and anti-HER2 agent trastuzumab emtansine was reported to overcome osimertinib resistance in T790M-positive EGFR-mutated NSCLC cell lines which gained HER2 amplification (24).

**Figure 1:**
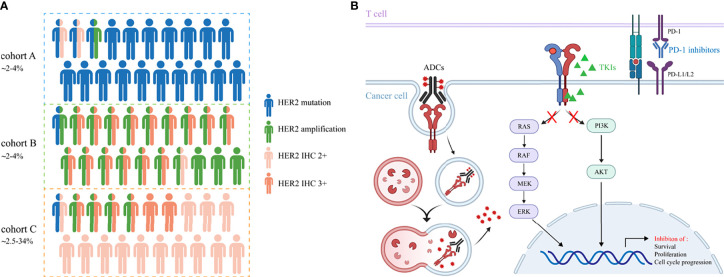
**(A)** The incidence rates and associations of HER2 alterations in NSCLC. Cohort A, B and C represent HER2 mutation, amplification and overexpression, respectively. Cohort C includes patients with IHC 2+ and 3+. **(B)** The underlying mechanisms of TKIs, ADCs and immune checkpoint inhibitors to cope with HER2-altered NSCLC.

The incidence rate of HER2 overexpression is reported with a wide range of 2.5%-34% in NSCLC, probably due to the inconsistency among different methods for positivity assessment and low concordance assessment by pathologists (9, 25–27). Currently, there is no consensus on how to define HER2 overexpression using IHC in NSCLC. Immunohistochemistry (IHC) scoring system and H-score are both applied for the assessment, with the former used more widely. Contrary to breast cancer, the co-occurrence of HER2 overexpression and amplification has not been well confirmed in lung cancer. However, an overlap between IHC 3+ staining and HER2 amplification was reported in NSCLC, albeit IHC low/negative has been also shown to be FISH positive. In the IHC 2+ cases, perhaps mechanisms other than gene amplification (HER2 polysomy, mutation and unknown reasons) cause the immuno-positive results (8, 26). Therefore, HER2 overexpression in NSCLC represents a heterogeneous group with distinct molecular features, making it a less accurate indicator of inhibitor sensitivities and patient outcomes. Further analyses need to be done to discriminate among these possibilities. The associations of HER2 mutation, amplification and overexpression are depicted in **Figure 1A**.

## Clinical Characteristics of HER2-Altered NSCLC

Mutations in HER2 are more frequent in females, never smokers and lung adenocarcinoma, similar to those observed in patients with EGFR mutations. However, HER2-mutated patients have a worse prognosis than their EGFR and ALK counterparts, partially due to the lack of highly-selective targeted agents (28–30). HER2 mutations also exhibit a tendency of brain metastases on treatment (31). Similarly, another study found that the HER2^YVMA^ subtype was associated with a higher estimated 12-month brain metastasis incidence compared with the non-YVMA group (40.2% vs. 3.6%, P=0.002) (32). *De novo* HER2 mutations in NSCLC are supposed to be mutually exclusive with other driver genes. But the oncogenic role of HER2 varied among different domains, with most of them occurring in the kinase domain. A study demonstrated that the frequency of EGFR or KRAS co-mutation was significantly higher in the non-TKD mutation compared to the TKD mutation, but OS was comparable between the two groups (28). Another retrospective database study revealed that patients harboring TMD mutations were diagnosed at more advanced stages (P<0.001) and had poorer OS (median OS 10.0m vs. 61.6m, P<0.001) than non-TMD mutations (33). TP53 aberrations were the most prevalent co-mutations in HER2-mutated patients, followed by aberrations in the PI3K/AKT/mTOR pathway. Both two co-mutation variants were correlated with shorter progression-free survival of afatinib treatment (11). This trend was also observed in another study which reported an impaired OS in patients with co-mutations in the cell-cycle pathway especially TP53 (34). These observations indicated different clinical implications of HER2 mutation variants and their co-mutations.

*De novo* HER2 amplification and HER2 overexpression were detected more often in smokers and male patients, implying their inconsistent origins of tumorigenesis with HER2 mutations (29, 35). HER2 amplification seemed to have a controversial role on the prognosis of NSCLC, while HER2 overexpression was a marker of poor prognosis, especially for adenocarcinoma, early-stage NSCLC and small cell lung cancer (SCLC) as suggested by a meta-analysis (36). Compared with their mutation variants, co-mutations of other driver genes were more frequently seen in HER2 amplification and overexpression tumors but still remained at a relatively low incidence rate. Concomitant HER2 alterations were proved to have an impact on the efficacy of targeted therapies. A retrospective study demonstrated that patients with concurrent EGFR mutation and HER2 amplification had a longer median time on treatment with EGFR-TKIs than those with EGFR mutation without HER2 amplification (846 days vs. 286 days, P =0.004) (37). However, in the post-hoc subgroup study of HER-CS, HER2 expression in EGFR-mutant patients may negatively impact the time-to-treatment failure (TTF) of EGFR-TKIs in the subgroup with a performance status (PS) of 2 (38).

## Targeting HER2 in NSCLC: Perplexity and Progress

### Chemotherapy and mAbs

Chemotherapy represents the most conventional treatment strategy of NSCLC patients with HER2 alterations before the arrival of targeted therapy. Several studies revealed no association between HER2 status and objective response to chemotherapy (27, 39). While another retrospective study reported the inferior outcomes of HER2-mutant NSCLC compared with ALK/ROS1-rearranged group, with a median PFS of 5.1 months in pemetrexed-based first-line chemotherapy. Additionally, subgroup analysis suggested that PFS had a trend to be inferior in the HER2^YVMA^ group compared with other variants, albeit not statistically significant (4.2 vs 7.2 months, P=0.085) (40). The retrospective study of the EUHER2 cohort showed the ORR and median PFS for patients receiving first-line conventional therapies (including chemotherapy and EGFR-TKIs) were 43.5% and 6 months. For those receiving second-line therapies, ORR and median PFS were 10% and 4.3 months (41). A Chinese study revealed that the median PFS and OS were comparable between first-line chemotherapy and HER2-targeted agents in HER2-mutant patients (5.9 vs 4.6 months, P=0.63; 9.8 vs 10.8 months, P=0.40, respectively) (42). While Xu et al. reported that compared with HER2-TKIs, chemotherapy achieved better outcomes both in the first-line and second-line setting. This trend was also observed in the HER2^YVMA^ subgroup (43).

Trastuzumab is a humanized IgG monoclonal antibody that could selectively inhibit the proliferation and survival of HER2-addictive tumors by targeting the extracellular domain of HER2 (44). A phase II study launched by the Eastern Cooperative Oncology Group (ECOG) evaluated the efficacy of combining carboplatin, paclitaxel and trastuzumab in advanced NSCLC patients with HER-2/neu positivity (1+ to 3+). The reported ORR, median PFS and median OS were 24.5%, 3.3 months and 10.1months, respectively. Notably, patients with 3+ HER-2/neu expression experienced a survival exceeding that of historical data, suggesting potential application for trastuzumab in this rare subgroup (45). Similarly, in a randomized phase II trial, the addition of trastuzumab to gemcitabine–cisplatin chemotherapy was beneficial for HER2 3+ or FISH-positive patients, but this subgroup is too small to provide definitive information (46). A phase IIa multiple basket study (Mypathway) also demonstrated a moderate efficacy of dual blockade with trastuzumab and pertuzumab in HER2-altered NSCLC patients (47). HOT1303-B trial was a multicenter, single-arm phase II study of trastuzumab for pretreated HER2-altered NSCLC patients, which were defined as HER2 mutations, IHC 3+ or IHC 2+/dual color *in situ* hybridization [DISH]+. Disappointingly, trastuzumab monotherapy did not produce any response (ORR was 0%) in this cohort, although DCR was 70.0% and the median PFS reached 5.2 months (48). A multicenter, phase II study (IFCT 1703-R2D2 trial) enrolled HER2-mutated advanced NSCLC patients progressing after platinum-based treatment and evaluated the efficacy and safety of triple therapy with trastuzumab, pertuzumab, and docetaxel (49). The ORR, median PFS and median OS were 29%, 6.8 months and 17.6 months, with tolerable toxicity, suggesting this triple therapy regimen becoming an option for pretreated patients.

In general, the efficacy of traditional chemotherapy for the HER2-altered population is far from satisfactory. The successes of trastuzumab observed in breast and gastric cancer might not be replicated in NSCLC. The reasons for differences in efficacy are complicated, but could possibly be explained by a different spectrum of HER2 alterations and other aspects of disease biology among cancer types. A combination of trastuzumab, pertuzumab, and docetaxel seemed to compete favorably with single mAb or chemotherapy, but this regimen remained to be defined. Other targeting strategies and agents for NSCLC patients with HER2 alterations are urgently needed.

### Non-Selective TKIs

Second-generation irreversible TKIs developed for the treatment of EGFR mutations represented early attempts to target HER2 in NSCLC. De Grève and colleagues reported the clinical benefits of afatinib in HER2 ex20ins LUAD patients (50). Among the five identified patients, three patients evaluable for the response all showed an objective response. Later on, the efficacy of afatinib was evaluated in a global named patient use program that enrolled HER2-mutant patients who had exhausted other treatments. Median TTF was 2.9 months, ORR and DCR were 19% and 69%, respectively. Notably, for the group of HER2^YVMA^ subtype, median TTF was 9.6 months and 40% continued treatment for more than one year (51). Conversely, other studies presented different perspectives, showing that the clonality status of HER2 ex20ins and the HER2^YVMA^ subtype were potential indicators for poor response to afatinib, while G778_P780dup and G776delinsVC subtypes derived favorable outcomes from afatinib, suggesting a further investigation into the clinical implications of mutation variants to help optimize outcomes with HER2-targeted therapies (11, 34). A prospective phase II NICHE trial exploring the efficacy of afatinib in pretreated advanced HER2-mutant NSCLC patients reported that ORR and DCR were 7.7% and 53.8%. Thus, the accrual into the trial was terminated with 13 patients enrolled altogether. Median PFS and OS were 15.9 and 56.0 weeks, respectively (52). Additionally, another phase II trial aimed to investigate the efficacy of afatinib among the Asian population with HER2-mutant NSCLC and consisted of two parts. In total, 18 patients were recruited and received afatinib in Part A. None of them achieved PR, 11 patients achieved SD and 6 patients had progressive disease as their best response. Median PFS and OS were 2.76 and 10.02 months, respectively. No patients met the Part B inclusion criteria, resulting in the termination of this study (53). The study of the EUHER2 cohort assessed the efficacy of chemotherapy and/or HER2-targeted agents in 101 advanced NSCLC patients with HER2 ex20ins. Sixty-five patients received HER2-targeted therapies. Eleven patients were treated with afatinib, with an ORR and median PFS of 18.2% and 3.9 months, respectively (41). Modest clinical activity of afatinib was also observed in a retrospective multicenter study, in which 27 patients included showed an ORR and median duration of response (DOR) of 13% and 6 months (54). A Chinese retrospective study included patients harboring HER2 mutation and amplification treated with afatinib and reported an ORR of 24%. Median PFS and OS were 3.3 and 13.9 months, respectively (55). Collectively, studies above showed inconsistent efficacy of afatinib for HER2-mutant patients. A meta-analysis study integrated and reanalyzed the existing data regarding afatinib treating HER2-mutant lung cancers. Pooled ORR and DCR were 21% and 66%, respectively, with the HER^YVMA^ subtype deriving greater clinical benefit (56). Thus, afatinib was not recommended as the regular application for treating NSCLC patients with HER2 mutation.

Dacomitinib is a pan-HER2 TKI that could bind to EGFR, HER2 and HER4 tyrosine kinases. A prespecified cohort from a phase II study enrolled NSCLC patients with HER2 mutations (n=26) or amplification (n=4). Three of 26 HER2-mutant patients had partial responses, but no partial responses were observed in four patients with HER2-amplified tumors. Median PFS and OS for HER2-mutant patients were 3 and 9 months, respectively. Interestingly, two patients harboring p. P780_Y781insGSP changes showed the longest responses, and the remaining patient with a partial response was identified with a p. M774delinsWLV mutation. No responses occurred in the most common HER2^YVMA^ subtype (57). Subsequently, a preclinical study demonstrated that the IC50 values of the HER2 ex20ins Ba/F3 cells harboring the dacomitinib-sensitive mutations (InsGSP, InsWLV, and InsCPG) were significantly lower than the other HER2 mutants or Wildtype HER2 (P=0.031), consistent with the previous clinical data (58).

Neratinib is another type of TKI which was applied and evaluated in HER2-mutant NSCLC. A preclinical *in vitro* study assessed the activity of drugs in HER2 mutation variants and found neratinib and afatinib more effective than other inhibitors for the HER2^YVMA^ subtype (59). A randomized 2-stage phase II study compared neratinib monotherapy and the combination of neratinib with temsirolimus in stage IIIB/IV HER2-mutant NSCLC patients. Twenty-seven patients were enrolled in stage 1, and 3 of 14 patients (21%) in the combination group had a response, resulting in a median PFS of 4 months (60). In the subsequent expansion cohort, the dual inhibition group obtained an ORR of 19%, a median PFS of 4.1 months and a median OS of 15.8 months. The incidence of grade 3 diarrhea was 12% and could be managed with loperamide prophylaxis (61). In the SUMMIT phase II basket trial, neratinib was evaluated across multiple cancer types. In patients with lung cancer (n=26), only one patient harboring L775S kinase missense mutation was observed with objective response (ORR=3.8%), suggesting its limited efficacy in HER2-mutated lung cancer. The median PFS in recurrent NSCLC was 5.5 months with 6 patients continuing therapy for more than one year (62).

The activities of non-selective TKIs in HER2-mutant NSCLC patients yield moderate or even disappointing results, though sporadic responses have been reported as HER2 mutation location could affect the drug binding affinity. This may be explained because HER2 ex20ins seem to tighten the drug-binding pocket, restricting the binding of large-sized inhibitors. Therefore, structurally novel pan-HER2 TKIs have been developed to achieve better outcomes in NSCLC with HER2 alterations.

### New-Generation TKIs

As a covalent and irreversible EGFR/HER2 inhibitor, poziotinib has a smaller size and flexible structure. A preclinical study compared the activity of different TKIs in Ba/F3 cells with HER2 exon 20 mutations, poziotinib showed the most potent activity. Also, the secondary C805S mutation was identified as a potential mechanism of acquired resistance to poziotinib (63). Another study indicated that HER2 20 exon insertions tightened the size of the drug-binding pockets and restricted the binding of large, rigid inhibitors using 3D modeling. Poziotinib can avoid these spatial changes owing to its small size and flexibility, thus becoming a potent inhibitor of the most common HER2 variant. Its efficacy was further confirmed *in vitro* and *in vivo* studies (64). Similar trends were also reported in the pan-cancer landscape and functional analysis of HER2 mutation by Robichaux and colleagues (12). In addition, in their preclinical models, poziotinib upregulated HER2 expression at the cell surface and potentiated the T-DM1 activity. Early results from a phase I study showed an encouraging activity of poziotinib and further justified its application in patients with EGFR/HER2 alterations (65). Based on this study and preclinical evidence, a phase II clinical trial of poziotinib in NSCLC patients with EGFR and HER2 exon 20 mutations was initiated. All HER2-mutant participants enrolled harbored the Y772dupYVMA or G778dupGSP insertions. Response was confirmed in 5 of 12 HER2-mutated patients (confirmed ORR=42%). The DCR and median PFS were 83% and 5.6 months. No patients discontinued treatment due to poziotinib-related toxicity (12). A poziotinib expanded access program enrolling NSCLC patients with EGFR or HER2 ex20ins showed a median PFS of 5.6 months and a median OS of 9.5 months. The ORR was higher in HER2 subgroup (50% vs. 23%). Grade 3 AEs were reported in 66% of the patients, and the toxicity rate was high leading to frequent dose interruption and reduction (66). A single-arm, open-label, phase II study assessed the efficacy and safety profiles of poziotinib in HER2-mutant advanced NSCLC. The confirmed ORR was 27% with responses observed across mutation subtypes. Median PFS and OS were 5.5 and 15.0 months, respectively. One possible treated-related death due to pneumonitis was reported (67). ZENITH20 study evaluated poziotinib in previously treated NSCLC patients with HER2 exon 20 insertions. In cohort 2, patients received poziotinib once daily, the ORR and DCR were 27.8% and 70.0%, respectively. Most patients (74%) had tumor reduction, with a median PFS was 5.5 months. Clinical benefits were seen regardless of types and lines of previous treatment, presence of brain metastasis and HER2 mutation variants. Severe treatment-related AEs (grade≥3) included rash (48.9%) diarrhea (25.6%), and stomatitis (24.4%) (68). Updating results from cohort 4 of ZENITH20 presented a promising efficacy in treatment-naive patients, with an ORR of 44% and a median PFS of 5.6 months, the safety profile was similar to cohort 2 of this study (69).

Pyrotinib is an oral, irreversible pan-HER TKI. The enhanced antitumor activity of another irreversible pan-HER TKI pyrotinib was observed in organoids as well as patients-derived xenograft (PDX) models relative to afatinib and trastuzumab-emtansine (T-DM1). In a phase II cohort of 15 HER2-mutant NSCLC patients, pyrotinib resulted in an ORR and a median PFS of 53.3% and 6.4 months. AEs were all grade 1-2 and no dose reduction or treatment discontinuation occurred (70). In another single-arm prospective study, pyrotinib exhibited promising efficacy and acceptable safety in 27 advanced HER2-amplified NSCLC patients, reaching a confirmed ORR of 22.2%, a median PFS of 6.3 months and a median OS of 12.5 months. Of note, Patients who received pyrotinib as a first-line treatment achieved a median PFS of 12.4 months. Treatment-related AEs occurred in all patients, but no grade 4 or higher AEs were documented (71). A larger phase II study evaluated the efficacy and safety profiles of pyrotinib in stage IIIB-IV LUAD patients harboring HER2 mutations after the failure of platinum-based chemotherapy. Independent reading committee-assessed ORR was 30.0%, with a favorable ORR observed across all HER2 subtypes and between patients with and without brain metastases (25.0% vs. 31.3%). The median PFS and OS were 6.9 and 14.4 months, respectively. Grade 3-4 treatment-related AEs occurred in 28.3% of patients and diarrhea (20.0%) appeared to be the most common type (72). In addition, the encouraging efficacy of pyrotinib was also reported in a study in which patients with EGFR mutation and HER2 amplification could obtain clinical benefits from combining EGFR-TKIs and pyrotinib, suggesting the potential of pyrotinib in targeting the HER2 pathway in patients after progressing on EGFR-TKIs (73).

The hypoxia-activated prodrug (HAP) tarloxotinib is a potential treatment for NSCLC with HER2 alterations. Prior work demonstrates that the tarloxotinib can be converted into tarloxotinib-E as its active form in a hypoxic tumor microenvironment. Preclinical studies showed that tarloxotinib-E could interfere with cell signaling and proliferation by inhibiting phosphorylation and activation of ERBB heterodimers in PDX models. *In vivo*, tarloxotinib inhibited tumor growth and progression. The pharmacokinetic analysis also confirmed the accumulations of tarloxotinib-E in tumor sites than plasma or skin (74). Another *in vitro* study demonstrated that the IC50 of tarloxotinib for wildtype HER2 was 180 times higher than that of tarloxotinib-E, suggesting a wide therapeutic index of tarloxotinib (75). The phase I RAIN-701 trial (NCT03805841) enrolled advanced patients with HER2 activating mutations (cohort B). First results showed that 2 of 9 patients experienced confirmed PR (22%) and 4 patients had SD. Grade 3 TEAEs included prolonged QTc (34.8%), increased ALT (4.3%), diarrhea (4.3%) and rash (4.3%) (76).

Mobocertinib (TAK-788) is a research-based oral EGFR/HER2 inhibitor designed against ex20ins. The IC50 of mobocertinib was higher than poziotinib and comparable with pyrotinib, neratinib and afatinib in HER2 ex20ins cell lines. Mobocertinib exhibited the lowest HER2 ex20ins IC50/wildtype EGFR IC50 ratio, implying its excellent selectivity profile. Additionally, lung cancers with HER2 G776delinsVC subtypes reported a superior response to mobocertinib than the YVMA subtypes. The combination of ado-trastuzumab emtansine (T-DM1) and mobocertinib had a synergistic function in HER2^YVMA^ tumors (77). A phase I/II trial (NCT02716116) showed promising antitumor activities of mobocertinib in advanced NSCLC patients harboring EGFR ex20ins, with a similar safety profile compared to other EGFR-TKIs (78, 79). Results from the expansion cohort 2 of this study which enrolled NSCLC patients with HER2 exon 20 alterations are still awaited.

Compared with non-selective TKIs originally developed for EGFR mutations, these novel TKIs have shown greater activities and broader anti-tumor effects across exons in HER2-mutant NSCLC. For patients who had previously received platinum-based chemotherapy where there are limited therapeutic drugs, new generation TKIs could be an option to consider.

### Antibody-Drug Conjugates

ADCs are characterized by the covalent coupling of drugs to mAbs as an alternative to naked antibody-targeted therapy. The development of ADCs represents groundbreaking progress in the treatment of malignancies with actionable targets. After their successes in breast and gastric cancer, these agents are gradually attracting much attention in NSCLC with HER2 alterations.

Trastuzumab emtansine (T-DM1) is a second-generation anti-HER2 ADC composed of trastuzumab and emtansine (DM1), which is an inhibitor of microtubule aggregation. This complex enters into HER2-positive cells *via* receptor-mediated endocytosis. Proteolytic degradation of the antibody moiety in lysosomes leads to the release of conjugated agents (80). In a phase II study of T-DM1 in relapsed HER2-positive NSCLC (IHC 3+, IHC 2+/FISH+, or exon 20 mutations), among fifteen assessable patients, only one patient achieved a PR (ORR=6.7%). The median PFS and OS were 2.0 and 10.9 months, respectively. Grade 3-4 AEs included thrombocytopenia (40%) and hepatotoxicity (20%), with no treatment-related deaths (81). However, another phase II basket trial enrolled 18 advanced LUAD patients harboring HER2 mutations. The treatment with T-DM1 might achieve an ORR as high as 44% and a median PFS of 5 months. Responses to T-DM1 could be seen across all the subtypes. The toxicities included grade 1-2 elevated hepatic transaminases, thrombocytopenia and infusion reactions (82). Peters et al. evaluated the efficacy and safety of T-DM1 in 49 advanced HER2-overexpressing NSCLC patients (29 IHC 2+ and 20 IHC 3+) who were previously treated. Although there were no treatment responses presented in the IHC 2+ cohort, four PR were observed in the IHC 3+ cohort (ORR=20%). Median PFS and OS were similar between the two cohorts (median PFS: 2.6 and 2.7 months; median OS: 12.2 and 15.3 months). Forty-five patients (92%) reported an AE of any grade and ten patients (20%) reported grade 3 AEs. One patient with a history of brain metastases reported grade 4 seizures while receiving seizure therapy. There were no deaths due to AEs (83).

Trastuzumab deruxtecan (T-Dxd, also known as DS-8201a) is a novel HER2-ADC composed of trastuzumab and a novel topoisomerase I inhibitor (MAAA-1181) linked by an enzymatically cleavable peptide. The drug moiety of T-Dxd could bind to topoisomerase I-DNA complexes and induce DNA double-strand breaks. T-Dxd has a drug-to-antibody ratio (DAR) of 8, which is almost two-fold higher than T-DM1 (DAR of 3-4). Preclinical results suggested that T-Dxd had antitumor activities towards a broad range of HER2-positive models and acceptable safety profiles. Featured by a highly membrane-permeable payload, it can exert the by-stander effect and is favorable in treating tumors that are insensitive to T-DM1. Thus, T-Dxd is expected to be a promising therapy to cope with HER2-positive or HER2-low-expressing tumors that do not respond to T-DM1 (84, 85). The first evidence of T-DM1 in NSCLC from a dose-expansion phase I study suggested that T-Dxd had great potential for HER2-expressing/mutant solid tumors. In the HER2-mutant/HER2-expressing NSCLC subgroup, 10 of 18 patients (55.6%) had a confirmed objective response, with a median PFS of 11.3 months. Among the subset of HER2-mutant NSCLC patients, the confirmed ORR even reached 72.7% (8/11) and the median PFS was 11.3 months. Notably, NSCLC patients with documented HER2 mutations had more pronounced tumor shrinkage than those without mutations, regardless of IHC status. All patients experienced at least 1 AE, and 2 of 18 patients (11.1%) had serious AEs. Three patients were adjudicated as interstitial lung disease (ILD) related to the study drug, and one patient experienced an AE of respiratory failure which was associated with a fatal outcome (86). The DESTINY-Lung01 phase II trial investigated the efficacy of T-Dxd in NSCLC patients and consisted of two cohorts. Cohort 1 contained patients with HER2 overexpression (IHC 2+ or IHC 3+), and cohort 2 contained patients with HER2 mutation. Results from cohort 1 showed an ORR and a median PFS of 24.5% and 5.4 months. Response rates were comparable according to HER2 IHC expression levels (ORR 25.6% vs. 20.0% in IHC2+ and IHC3+ patients, respectively). All patients had at least one treatment-emergent AEs, and grade 3 AEs were reported in 73.5% of patients. There were 8 cases of drug-related ILD as adjudicated by an independent committee. Treatment-emergent AEs were associated with dose interruption in 26 patients (53.1%), dose reduction in 17 patients (34.7%), and treatment discontinuation in 11 patients (22.4%) (87). Recently published results from cohort 2 were promising and more spectacular in comparison with cohort 1. Among 91 patients enrolled, centrally confirmed ORR was 55%, the median PFS was 8.2 months and the median OS was 17.8 months (88). Preclinical findings revealed that HER2-activating mutations facilitated receptor-mediated endocytosis of the HER2-ADC complex (89). This may provide the mechanistic foundation for its higher efficacy in HER2-mutant NSCLC patients in contrast to the lower response rates among HER2-overexpressing patients. Regarding T-Dxd toxicity, common events included gastrointestinal and hematologic events, decreased appetite, and alopecia. Grade 3 or higher drug-related AEs occurred in 42 patients (46%). Twenty-three patients (25%) discontinued treatment because of investigator-reported, drug-related AEs, including pneumonitis in 12 patients and ILD in 5 patients (88). Given the exciting results from DESTINY-Lung01, a randomized, open-label, phase 3 trial (DESTINY-Lung04; NCT05048797) is underway to further evaluate the efficacy and safety of T-Dxd compared to standard of care (pembrolizumab combined with chemotherapy) in non-squamous NSCLC patients harboring a HER2 exon 19 or 20 mutations.

By far, ADC-based therapies seem to provide the highest response rates and best clinical outcomes among the anti-HER2 agents, both in HER2-mutant and HER2-overexpressed NSCLC patients. However, drug-related ILD is observed during the treatment and further studies should be carried out to determine which patients are at great risks and how to manage this potentially fatal AE.

### Immunotherapy

Immune checkpoint inhibitors (ICIs) demonstrated significant improvements in overall response and survival for driver-negative NSCLC patients, while their application in oncogene-addicted tumors remains to be elucidated. The presence of HER2 mutations in NSCLC is correlated with a “cold” tumor microenvironment, characterized by a relatively lower PD-L1 positive expression rate and tumor mutation burden (TMB), similar to those of EGFR-dependent tumors (90, 91). The efficacy of ICIs monotherapy was evaluated in several studies. A retrospective study identified 26 patients who were treated with ICIs. ORR was 12% (3/26), the median PFS and OS were 1.9 and 10.4 months, respectively. Of the three responders, none had a HER2 YVMA mutation, two had PD-L1 ≥50%, and two had TMB ≥median (92). In the IMMUNOTARGET registry, 29 NSCLC patients with HER2 mutation received ICIs and the ORR was 7%. The median PFS was 2.5 months and was significantly associated with positive smoking status (3.4 months in smokers vs. 2 months in non-smokers, P=0.04) (93). The French Lung Cancer Group (GFPC) reported 6 out of 23 relapsed HER2-mutant NSCLC patients had objective responses to ICIs, with a median DOR of 15.2 months. Survival data were close to previous reports, with a median PFS of 2.2 months and a median OS of 20.4 months (94). Other studies also showed moderate or even dismal responses and survival outcomes of ICIs monotherapy against HER2 mutations (90, 91, 95).

Considering the synergistic antitumor effects of combining ICIs and chemotherapy, ICIs-based therapies were explored and been evaluated. A multicenter retrospective study enrolled 26 HER2-mutant NSCLC patients, most of which received immunochemotherapy combination regimens. The ORR, DCR and median PFS were 38.5%, 84.6% and 7.4 months, respectively (96). Additionally, results from another study demonstrated that the ORR, median PFS, and one-year OS rate of ICIs combined with chemotherapy for treatment-naive HER2-mutant NSCLC were 52%, 6 months and 88%, respectively (97). Tian et al. reported similar clinical outcomes of chemo-immunotherapy in 13 stage IV HER2 ex20ins LUAD patients, with an ORR of 31% and a median PFS of 8.0 months. They also found a higher TMB, a trend toward lower clonality of tumors and a trend toward lower TCR diversity of peripheral blood in responders compared with non-responders (P=0.0067, 0.071 and 0.085, respectively). Patients with baseline TMB-high combined with mutations in DNA damage repair-related pathways or SWI/SNF complex was associated with favorable outcomes of chemo-immunotherapy combinations (98).

Evidence on the efficacy of ICIs in HER2-mutant NSCLC patients remains limited and is heavily derived from retrospective results. These studies do not encourage the use of ICIs monotherapy as a therapeutic strategy in HER2-mutant NSCLC patients. Immunotherapy-based approaches could be a potential treatment option for this patient group and further clinical trials are required to confirm these results. Clinical trials of anti-HER2 agents in NSCLC patients mentioned in this review are summarized in **Table 1**. Promising strategies to cope with HER2-altered NSCLC and their underlying mechanisms are shown in **Figure 1B**.

**Table 1:** Clinical trials of anti-HER2 agents in NSCLC patients.

References	Agents	Clinical trials	N	Population	HER2 alterations	ORR n (%)	Median PFS months, (95% CI)	Median OS months, (95% CI)
Langer et al. (45)	Trastuzumab + CT	Phase II study	53	Recurrent, Stage IIIB/IV NSCLC	HER2 positivity (1+ to 3+)	13 (24.5)	3.3 (NA)	10.1 (6.7-14.6)
Gatzemeier et al. (46)	Trastuzumab + CT	Phase II study	50	Untreated stage IIIB/IV NSCLC	HER2 IHC 2+/3+ or serum HER2 ECD positive	18 (36)	6.1 (0.1-19.6)	12.2 (0.1-19.6)
Hainsworth et al. (47)	Pertuzumab + Trastuzumab	Phase IIa basket study (MyPathway)	16	Refractory, metastatic NSCLC	HER2 amplification or overexpression	2 (13)	NA	NA
			14		HER2 mutation	3 (21)	NA	NA
Kinoshita et al. (48)	Trastuzumab	Phase II study (HOT1303-B)	10	NSCLC patients pretreated with ≥2 regimens	HER2 IHC 3+, IHC2+/DISH+ or mutation	0 (0)	5.2 (1.4-6.3)	NA
Mazieres et al. (49)	Pertuzumab + Trastuzumab + Docetaxel	Phase II study (IFCT-1703 R2D2)	45	Advanced NSCLC, progressed after ≥1 platinum-based treatment	HER2 mutation	13 (29)	6.8 (4.0-8.5)	17.6 (11.6-18.9)
Peters et al. (51)	Afatinib	Global Named Patient Use Program	28	Heavily pretreated, stage IV NSCLC	HER2 mutation	3/16 (19)^a^	NA	NA
Dziadziuszko et al. (52)	Afatinib	Phase II study (NICHE)	13	Pretreated, advanced NSCLC	HER2 mutation	1 (7.7)	3.7 (1.4-8.3)	13.1 (3.8-NE)
Fan et al. (53)	Afatinib	Phase II study	18	Pretreated, advanced NSCLC	HER2 mutation	0 (0)	2.76 (1.87-4.60)	10.02 (8.47-10.08)
Kris et al. (57)	Dacomitinib	Phase II study	26	Advanced NSCLC, 83% pretreated with CT	HER2 mutation	3 (11.5)	3 (2-4)	9 (7-21)
			4		HER2 amplification	0 (0)	1,1,5,5	5,7,15,22
Besse et al. (60)	Neratinib (N)±Temsiromilus (TEM)	Phase II study	13	Stage IIIB/IV NSCLC (N)	HER2 mutation	0 (0)	2.9 (1.4-NE)	NA
			14	Stage IIIB/IV NSCLC (N + TEM)		3 (21)	4.0 (2.9-9.8)	
Gandhi et al. (61)		Phase II study (Expansion cohort)	17	Stage IIIB/IV NSCLC (N)	HER2 mutation	0 (0)	3.0 (1.4-6.9)	10.0 (4.9-19.0)
			43	Stage IIIB/IV NSCLC (N + TEM)		8 (19)	4.1 (2.9-5.6)	15.8 (10.8-19.5)
Hyman et al. (62)	Neratinib	Phase II basket study (SUMMIT)	26	Pretreated, advanced NSCLC	HER2 mutation	1 (3.8)	5.5 (NA)	NA
Robichaux et al. (12)	Poziotinib	Phase II study	12	Metastatic, recurrent NSCLC	HER2 mutation	5 (42)	5.6 (NA)	NA
Prelaj et al. (66)	Poziotinib	Phase II study	8^b^	Advanced NSCLC	HER2 mutation	4 (50)	5.6 (3.6-6.7) ^c^	9.5 (5.3-NE) ^c^
Elamin et al. (67)	Poziotinib	Phase II study	30	Stage IV or recurrent NSCLC, 90% of patients were pretreated	HER2 mutation	8 (27)	5.5 (4.0-7.0)	15 (9.0-NE)
Le et al. (68)	Poziotinib	Phase II study (ZENITH 20)	90	Pretreated, advanced NSCLC	HER2 mutation	25 (27.8)	5.5 (3.9-5.8)	NA
Cornelissen et al. (69)			48	Treatment naïve, advanced NSCLC	HER2 mutation	21 (44)	5.6 (NA)	NA
Wang et al. (70)	Pyrotinib	Phase II study	15	Pretreated, advanced NSCLC	HER2 mutation	8 (53.3)	6.4 (1.60-11.20)	12.9 (2.05-23.75)
Song et al. (71)	Pyrotinib	Prospective, single-arm trial	27	Stage IIIB/IV NSCLC	HER2 amplification	6 (22.2)	6.3 (3.0-9.6)	12.5 (8.2-16.8)
Zhou et al. (73)	Pyrotinib	Phase II study	60	Pretreated, advanced NSCLC	HER2 mutation	18 (30)	6.9 (5.5-8.3)	14.4 (12.3-21.3)
Hotta et al. (81)	T-DM1	Phase II study	15	Pretreated, advanced NSCLC	HER2 IHC 3+, IHC2+/FISH+ or mutation	1 (6.7)	2.0 (1.4-4.0)	10.9 (4.4-12.0)
Li et al. (82)	T-DM1	Phase II basket study	18	Advanced NSCLC, 83% pretreated with CT	HER2 mutation	8 (44)	5 (3-9)	NA
Peters et al. (83)	T-DM1	Phase II study	29	Locally advanced or metastatic NSCLC, pretreated with ≥1 CT	HER2 IHC 2+	0 (0)	2.6 (1.4-2.8)	12.2 (3.8-23.3)
			20		HER2 IHC 3+	4 (20)	2.7 (1.4-8.3)	15.3 (4.1-NE)
Tsurutani et al. (86)	T-DXd	Phase I study	18	Pretreated, advanced or recurrent NSCLC	HER2 overexpression or mutation	10 (55.6)	11.3 (7.2-14.3)	NR (17.3-NE)
Nakagawa et al. (87)	T-DXd	Phase II study (DESTINY-Lung01)	49	Pretreated, metastatic NSCLC	HER2 overexpression	12 (24.5)	5.4 (2.8-7.0)	NA
Li et al. (88)			91	Pretreated, unresectable or metastatic NSCLC	HER2 mutation	50 (55)	8.2 (6.0-11.9)	17.8 (13.8-22.1)

N, number; ORR, objective response rate; PFS, progression-free survival; OS, overall survival; CT, chemotherapy; IHC, immunohistochemistry; ECD, extracellular domain; DISH, dual color in situ hybridization; FISH, fluorescence in situ hybridization; NA, not available; NE, not estimable; NR, not reached.

a Tumor response data were available for 16 patients. ^b^This phase II study enrolled 30 patients, 22 had EGFR 20 exon mutations and 8 had HER2 mutations. ^c^PFS and OS data were evaluated based on the whole cohort (n=30).

## Discussion

HER2 alterations, including mutation, amplification and overexpression, have emerged as novel potential targets for anti-HER2 agents in NSCLC. Following new drug designations rapidly changing the treatment landscape of this particular subset of NSCLC patients, several matters still need to be discussed and addressed.

First of all, the inconsistency in the clinical efficacy of anti-HER2 drugs among HER2 variants implies their distinct molecular entities and features. Currently, most studies evaluating anti-HER2 agents in NSCLC patients are relatively small-sampled and do not distinguish the three types of HER2 alterations. Thus, it leaves us the question of how we define the term ‘HER2-positive’ and which types of ‘HER2-positive’ lung cancers are suitable for receiving anti-HER2 targeted therapy. As mentioned above, apart from breast and gastric cancers, the patterns of HER2 staining in other cancer types have not been thoroughly investigated. This probably explains the variations of IHC overexpression rates reported in different studies. Consequently, IHC expression is not a definitive biomarker for anti-HER2 activity in NSCLC. In order to define HER2 alterations and determine their response patterns, it is crucial to advocate for standardized methods in the future to establish the definitive formulation of HER2 activating mutation, amplification and overexpression. Patient cohorts enrolled for evaluating HER2-targeted drugs should also be distinguished by the particular HER2 alteration present.

Secondly, given the substantial variant efficacy and safety profiles of anti-HER2 drugs, it is imperative to investigate the pharmacodynamic and pharmacokinetic properties of these agents. Studies also reported that HER2-mutated NSCLCs are associated with central nervous system (CNS) metastases during treatment in approximately half of the patients. Therefore, frequent monitoring for the early identification of CNS involvement along with the attentive assessment of the intracranial activity of both existing and forthcoming anti-HER2 drugs are of great importance.

Thirdly, most of the current studies focused on the efficacy of single anti-HER2 agents. But preclinical studies have demonstrated the additional or synergistic effects of combining ADCs with irreversible TKIs or immunotherapy. Li et al. reported that co-treatment with irreversible pan-HER inhibitors promoted receptor ubiquitination and consequent internalization of ADC complex, resulting in improved efficacy. Switching from T-DM1 to T-Dxd, which exhibited a different cytotoxic payload, could achieve durable responses after developing resistance to T-DM1 (89). Moreover, T-Dxd increased tumor-infiltrating CD8+ T cells and enhanced the expression of PD-L1 and MHC-I on tumor cells. Combining T-Dxd and PD-1 inhibitors is more effective than single-agent monotherapy in mouse models. These findings provide evidence for the rationale of combination therapy in patients with HER2-altered NSCLC (99). Therefore, a paradigm shift from monotherapies towards combination therapies will probably change the current treatment landscape for HER2-addicted NSCLC. ADC-based therapies seem to provide the highest response rates and the best survival outcomes in HER2-altered NSCLC patients by far. Several clinical trials (NCT03334617, NCT04686305) have been launched to address this issue. Also, with more agents in the pipeline are waited to be approved, the sequencing of these novel therapies is attracting more attention.

Another issue is the not-infrequent situation of concomitant mutations in HER2-altered NSCLC patients (for example, TP53), which can impair the efficacy of anti-HER2 agents and affect prognosis. Furthermore, acquired HER2 mutation and amplification are considered to be the main mechanisms of bypass pathways driving EGFR-TKIs resistance, suggesting the expansion of TKIs-induced selection of HER2-driven cell clones. Under these circumstances, concomitant treatment of EGFR-TKIs and HER2-targeted agents could become a promising therapeutic strategy. Further investigations are needed to provide the rationale for targeting HER2 in these clinical settings.

Not long ago, HER2 alterations were considered poor targets in NSCLC and were deemed to have inferior clinical outcomes than those with other genetic alterations. Nowadays, owing to advances in preclinical biology-based drug development, the wide array of the investigating HER2-targeted agents is a glimmer of hope shooting through the past prejudice. With emerging therapies requiring further clinical validation, exploration of targeting HER2 in NSCLC is underway.

## Author Contributions

Manuscript writing: XY, XJ. Manuscript revision: CS. Read and approve the final manuscript: XY, XJ, CS. All authors contributed to the article and approved the submitted version.

## Funding

This study was supported by the National Natural Science Foundation of China (grant numbers: 81874036, 82072568), the Science and Technology Commission of Shanghai Municipality (grant number: 19411971100) and the Shanghai Shenkang Hospital Development Center (grant number: SHDC12020110).

## Conflict of Interest

The authors declare that the research was conducted in the absence of any commercial or financial relationships that could be construed as a potential conflict of interest.

## Publisher’s Note

All claims expressed in this article are solely those of the authors and do not necessarily represent those of their affiliated organizations, or those of the publisher, the editors and the reviewers. Any product that may be evaluated in this article, or claim that may be made by its manufacturer, is not guaranteed or endorsed by the publisher.
